# Reducing Loneliness and Social Isolation of Older Adults Through Voice Assistants: Literature Review and Bibliometric Analysis

**DOI:** 10.2196/50534

**Published:** 2024-03-18

**Authors:** Rachele Alessandra Marziali, Claudia Franceschetti, Adrian Dinculescu, Alexandru Nistorescu, Dominic Mircea Kristály, Adrian Alexandru Moșoi, Ronny Broekx, Mihaela Marin, Cristian Vizitiu, Sorin-Aurel Moraru, Lorena Rossi, Mirko Di Rosa

**Affiliations:** 1 Centre for Innovative Models for Aging Care and Technology, IRCCS INRCA-National Institute of Health and Science on Aging Ancona Italy; 2 The Space Applications and Technologies Laboratory, Institute of Space Science – Subsidiary of INFLPR (National Institute for Laser, Plasma and Radiation Physics) Magurele Romania; 3 Department of Automatics and Information Technology, Faculty of Electrical Engineering and Computer Science, Transilvania University of Brasov Brasov Romania; 4 Department of Psychology and Education Sciences, Faculty of Psychology and Education Sciences, Transilvania University of Brasov Brasov Romania; 5 Innovation Department, ePoint Hamont Belgium; 6 Centre for Biostatistics and Applied Geriatric Clinical Epidemiology, IRCCS INRCA-National Institute of Health and Science on Aging Ancona Italy

**Keywords:** voice assistant, loneliness, social isolation, older adults, literature review, bibliometric analysis, mobile phone

## Abstract

**Background:**

Loneliness and social isolation are major public health concerns for older adults, with severe mental and physical health consequences. New technologies may have a great impact in providing support to the daily lives of older adults and addressing the many challenges they face. In this scenario, technologies based on voice assistants (VAs) are of great interest and potential benefit in reducing loneliness and social isolation in this population, because they could overcome existing barriers with other digital technologies through easier and more natural human-computer interaction.

**Objective:**

This study aims to investigate the use of VAs to reduce loneliness and social isolation of older adults by performing a systematic literature review and a bibliometric cluster mapping analysis.

**Methods:**

We searched PubMed, Embase, and Scopus databases for articles that were published in the last 6 years, related to the following main topics: voice interface, VA, older adults, isolation, and loneliness. A total of 40 articles were found, of which 16 (40%) were included in this review. The included articles were then assessed through a qualitative scoring method and summarized. Finally, a bibliometric analysis was conducted using VOSviewer software (Leiden University’s Centre for Science and Technology Studies).

**Results:**

Of the 16 articles included in the review, only 2 (13%) were considered of poor methodological quality, whereas 9 (56%) were of medium quality and 5 (31%) were of high quality. Finally, through bibliometric analysis, 221 keywords were extracted, of which 36 (16%) were selected. The most important keywords, by number of occurrences and by total link strength; results of the analysis with the Association Strength normalization method; and default values were then presented. The final bibliometric network consisted of 36 selected keywords, which were grouped into 3 clusters related to 3 main topics (ie, VA use for social isolation among older adults, the significance of age in the context of loneliness, and the impact of sex factors on well-being). For most of the selected articles, the effect of VA on social isolation and loneliness of older adults was a minor theme. However, more investigations were done on user experience, obtaining preliminary positive results.

**Conclusions:**

Most articles on the use of VAs by older adults to reduce social isolation and loneliness focus on usability, acceptability, or user experience. Nevertheless, studies directly addressing the impact that using a VA has on the social isolation and loneliness of older adults find positive and promising results and provide important information for future research, interventions, and policy development in the field of geriatric care and technology.

## Introduction

### Background

Nowadays, the aging of the population presents new challenges that requires consideration and response [1]. Among the major public health concerns regarding older adults, 2 significant concerns are loneliness and social isolation [2].

In fact, social networks seem to decrease with age and the prevalence of loneliness is estimated to increase as the population ages [2], to the extent that Valtorta and Hanratty [3] define loneliness and isolation as being “increasingly part of the experience of growing old.”

Social isolation and loneliness have severe consequences for older adults’ mental and physical health, including depressive symptoms [4], dementia [5], coronary heart disease and stroke [6], and mortality [7]. Moreover, social isolation and loneliness also have adverse outcomes concerning the use of health services, increasing emergency department and physician visits, hospital readmissions, and long-term care admissions [8].

New technologies may have a great impact on providing support in the daily lives of older people, especially in the areas of health monitoring, security, and comfort [9]. Therefore, they could be valuable tools to respond to the many challenges that older adults face.

In this scenario, technologies based on voice assistants (VAs) are of great interest and have potential benefits. VAs are systems based on artificial intelligence techniques that are programmed to be activated at a specific wake word to capture the user’s voice, process and interpret the command via a server, and respond back with a voice response or completed task [10].

**Figure 1:**
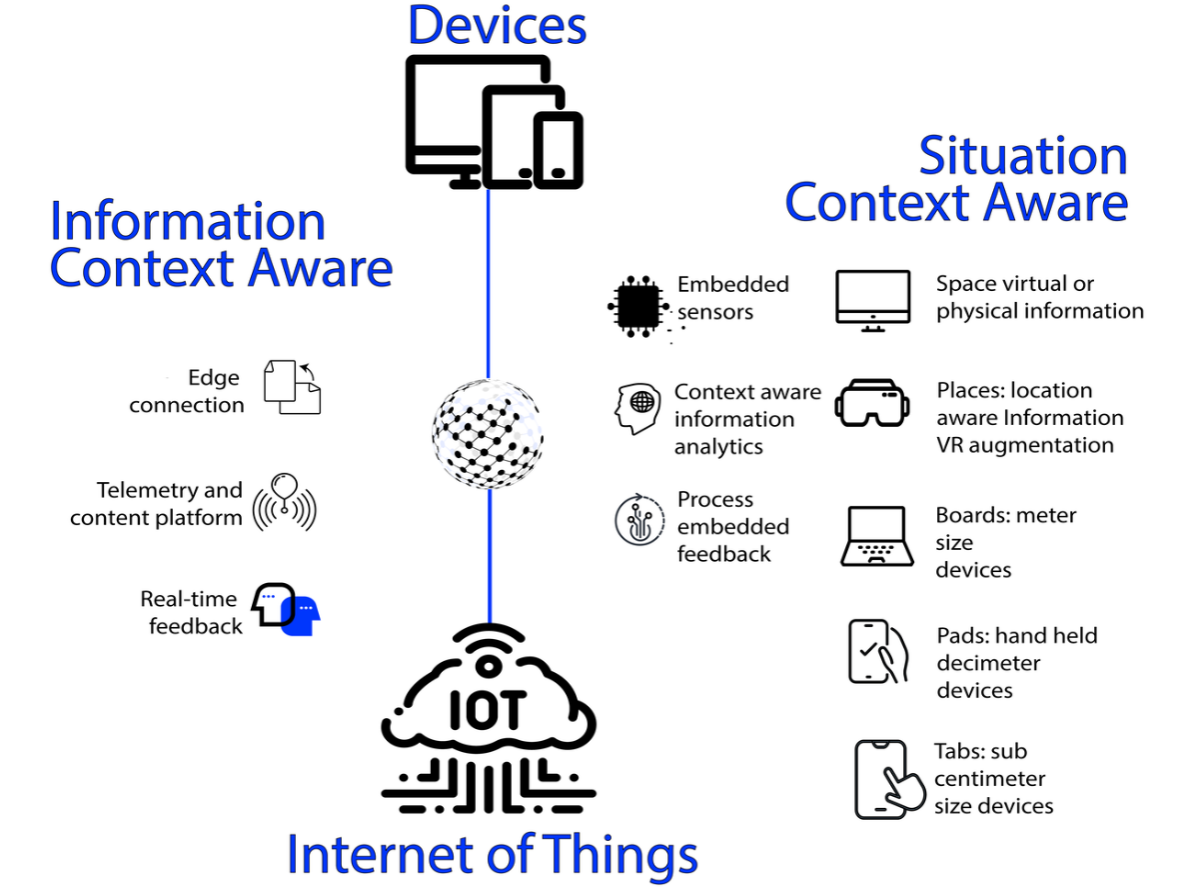
The functioning of ubiquitous computing. VR: virtual reality.

VA systems have the potential to support behavioral interventions using everyday life technologies such as smartphones, tablets, and smart speakers [9]. The strength behind the use of voice-based technology, having reached a significant stage of maturity, is strictly related to the concept of *ubiquitous computing* (Figure 1), introduced by Weiser in 1991 when thinking about a paradigm of technology able to adapt to the human environment that *vanish in the background* [11]. Indeed, VA technology is physically intangible; it does not force the user to be physically at a particular place to operate, and it provides interaction using natural language [9].

Concerning the application to older people, this easy and natural human-computer interaction gives VA systems the potential to overcome possible barriers existing with other digital technologies, which appears particularly promising and appropriate [9].

### Objectives

In light of this, the objective of this study is to investigate the use of VAs to reduce loneliness and social isolation of older adults by performing a literature review and a bibliometric analysis.

## Methods

### Database Creation

A literature search of scientific articles published from January 1, 2018, to April 4, 2023, was conducted. Considering that VA technology had not reached a significant stage of maturity, especially in its application for social purposes, this time range was defined.

The PubMed, Embase, and Scopus databases were searched to extend the range of eligible articles. In particular, the search was performed by setting up the “Title/Abstract” field in PubMed, the “Title or Abstract” field in Embase, and the “Title, Abstract, Keywords” field in Scopus.

The search was performed using an appropriate sequence of keywords, based on the research objectives. The first part of the search string was focused on synonyms for VA, whereas the second part specified the application for isolation and loneliness in older adults. The search string used was as follows: ((voice interface) OR (voice assistant) OR (vocal interface) OR (vocal assistant) OR (speech agent) OR (vocal agent)) AND (olde* OR elder*) AND (isolation OR loneliness).

We collected a total of 40 publications: 34 from Scopus, 4 from PubMed, and 2 from Embase.

### Study Selection

The selection of the eligible studies was performed according to the following principles:

1. Including only publications in English language: no documents were excluded.
2. Removal of overlaps between the different databases: 3 overlapping documents were identified.
3. Excluding papers in which the title and abstract were not relevant to the research question: 12 papers were excluded.
4. Removal of articles not retrieved: 1 article was excluded.
5. Excluding articles not pertinent to the research question: 8 documents were excluded.

The studies were assessed independently by 3 authors (CF, RAM, and AD). Any disagreement and uncertainties in the study selection were resolved by discussion. In particular, 2 authors conducted the first assessment, and another one solved the divergences.

Multimedia Appendix 1 [12-19] reports the list of excluded articles concerning eligibility assessment and details about the motivations for their exclusion.

The final database was composed of 40% (16/40) of the collected documents.

**Figure 2:**
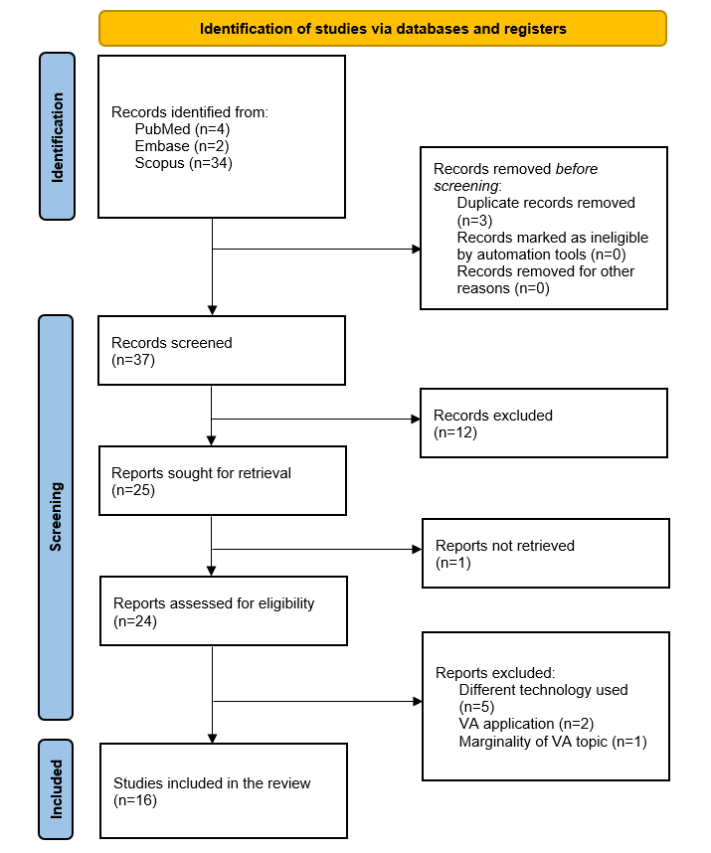
PRISMA (Preferred Reporting Items for Systematic Reviews and Meta-Analyses) flow diagram. VA: voice assistant.

Figure 2 reports the PRISMA (Preferred Reporting Items for Systematic Reviews and Meta-Analyses) flow diagram [20], summarizing the identification, screening, and inclusion procedures performed.

### Quality Scoring

As systematic reviews are comprehensive and rigorous assessments of existing literature on a specific research question and they aim to synthesize the available evidence to provide a reliable and unbiased summary, the “Tool for Scoring Quality of Non-Empirical Data Sources” [21], owned by the Aerospace Medicine Systematic Review Group, was used to assess the quality of individual studies included in this review. In total, 2 authors (RAM and CF) performed this evaluation independently, solving any disagreements or doubts through discussion. It is important to note that the purpose of quality scoring in systematic reviews is not to exclude studies but rather to provide an evaluation of their methodological strengths and weaknesses. The scoring process helps reviewers assess the overall risk of bias in the body of evidence and inform their conclusions and recommendations.

### Data Extraction

To perform the synthesis of findings, a data extraction from the 16 selected articles was conducted. The extraction consisted of a further evaluation of the full text of the articles. In total, 2 authors (MDR and CF) independently extracted information from the selected studies, including reference, population, technological solution, environment, study design, outcomes, and main results. The assessors made the information homogeneous and analyzed the articles together in the case of doubts or missing data. The data extracted were reported in the corresponding section of the synthesis of findings table (Table 1).

**Table 1:** Synthesis of findings.

Study, year	Population	Technological solution	Environment	Study design	Outcomes	Main results
Balasubramanian et al [22], 2021	A total of 44 adults (2 types of cohorts: one specifically focused on diabetes and the other on a range of long-term health conditions such as multiple sclerosis, dementia, and depression) and 7 informal carers, with age ranged from 50 to 90 y	VA^a^ commercial device (Alexa Echo Show 8)	Home	Qualitative (service evaluation and market research for a pilot service redesign program)	Acceptance and user experience about assistive technology for health and social well-being	Positive impact on the health and social well-being of the users; many direct and indirect benefits were identified: reminders for medications and appointments; improved adherence and disease control; increased independence and productivity; and, for those living alone, the device helped combat their loneliness and low mood
Bravo et al [23], 2020	A total of 10 older adults (aged 60 to 77 y)	VA embedded in a mobile app	Retirement home or home with their family	Quantitative (evaluation test)	VA evaluation: naturalness, embodiment, interaction and affect, joy of use, ease of use, acceptability, and utility (5-point Likert scale)	Average satisfaction score of 3.98 on a scale of 1 (lowest) to 5 (highest; naturalness: 3.8, embodiment: 3.73, interaction and affect: 4.18, joy of use: 3.99, ease of use: 4.03, acceptability: 3.68, and utility 4.29)
Caselgrandi et al [24], 2021	A total of 39 adults aged >50 y (mean 63; 78% male adults) with PACS^b^, previously hospitalized for severe COVID pneumonia	VA commercial device (Google Nest)	Home	Quantitative (pre-post study)	Assessment of clusters of PACS symptoms, frailty, and HRQoL^c^: depression, anxiety, and stress (DASS-21^d^); resilience (CD-RISC-25^e^), frailty (Reference Sites Network for Prevention and Care of Frailty and Chronic Conditions in community dwelling persons of EU Countries—SUNFRAIL), quality of life (EQ-5D-5L^f^), insomnia (ISI^g^), health (SF-36^h^); frailty phenotype; assessment for sarcopenia (hand grip measured with dynamometer); satisfaction with the VA tool; patients’ empowerment: proportion of people acquiring a normal caloric and normal protein diet, reduction of sedentary life, and engagement with moderate and vigorous physical activity	PACS cluster symptoms, frailty, and HRQoL improved at 6 mo follow-up; 96% of the participants considered VA useful; 44% used VA for entertainment and to cope with loneliness; and 48% of the participants modified sedentary life
Corbett et al [25], 2021	Older adults	VAs Commercial devices (eg, Amazon Echo and Google Nest)	Home	Mini review	Social isolation and loneliness	VAs are perceived by many older adult users as “companions” and improve social connectedness and reduce loneliness
Farías-Barraza et al [26], 2022	A total of 1 adult (female; alpha tests) and 6 older adults aged >65 y (3 female adults and 3 male adults) with no experience with VA technology (beta tests)	VA with open-source software and available technologies (PC application)	Laboratory	Quantitative (development tests [alpha tests] and users test [beta tests])	Right answer ratio and percentage for all 20 commands (alpha tests). Functions success percentage; key indicators of success: language, right answers, app cost, and 20 basic existing commands (beta tests)	Alpha test: in all the commands, right answer ratio percentage >75% (lowest percentages given in “Timer” and “Conversation” functions). Beta test: language, app cost, 20 basic existing commands and right answers: achieved (average number of correct answers 86.9% and conversation function within the fewest values)
García-Méndez et al [27], 2021	A total of 31 older adults (20 female adults and 11 male adults; mean 75.5, SD 6.95 y; 10 had some basic technology skills [such as experience with Google or WhatsApp]) and 8 had hearing problems	VA developed as a chatbot (tablet) implementing Google Voice Android Software Development Kit	Laboratory	Mixed (evaluation test)	Users’ experience: satisfaction, amazement, and chatbot-human likeliness (5-level Likert scale); users’ ability to describe the content presented (NGD^i^) between news metadata and the words users explicitly chose to search for news; knowledge about the news in the dialogue between the chatbot and the users (NGD); and liked or disliked aspects	Users’ satisfaction with the service close to 4 and the perception of chatbot-human likeliness close to 3 on average. Describing presented content, focused users with technology skills selected effective words (NGD=0.56), whereas stressed or confused users provided vague terms (NGD=0.84). Visual indications of the user’s turn to speak, chatbot empathy, frontend avatar, and newscaster functionality were praised. Confused users were particularly baffled by chatbot interruptions when they paused for too long
Jones et al [28], 2021	A total of 16 older adults (69% female adults and 31% male adults) aged >75 y (mean 85.2, SD 5.02 y) with normative cognitive functioning	VA commercial device (Amazon Echo)	Independent living facility	Mixed (single-group quasi-experimental study)	Perceived loneliness (8-item University of California, Los Angeles Loneliness Scale) and anthropomorphic aspects of the interactions (relational greetings, comments and questions, polite interactions, and reaction)	Significant reductions in perceived loneliness after 4 wk of using the VA. Relational greetings to the Alexa VA predicted 4-wk loneliness reductions, whereas the number of reactions, polite interactions, or comments and questions did not significantly predict 4-wk loneliness reductions
O’Brien et al [29], 2022	A total of 16 adults (14 female adults and 2 male adults): 11 geriatric experts aged >21 y and 5 older adults aged >65 y	VA Commercial device (Google Home)	Home	Qualitative (evaluation test)	Experience with the device; codes and overarching themes	A total of 288 comments were received from which 8 major themes were identified as possible beneficial functions of VA: administrative, companionship, home control, education, emergencies, entertainment, health and well-being, and reminders
Pech et al [30], 2022	A total of 109 adults (86 female adults and 23 male adults) aged >50 (mean 81.2, SD 8.6) y with no severe visual or hearing impairment and no moderate to severe cognitive impairment	Digital intelligent platform available on smartphone, tablet, or computer and a VA	Home	Mixed (pre-post study)	System use and acceptability, service use and satisfaction, intervention global perception, system improvement, and operational team’s feedback	A total of 39% used the services at least once; 63% had a positive opinion toward the system; 22% had a positive opinion on the intervention, 55% a mixed opinion and 23% a negative one; proposed improvements were easy access to trusted professionals, communication about city events, late-night pharmacy, activity propositions tailored to their needs, and videoconferencing option; and the team emphasized older adults’ resistance to change, unplanned workload, and specific technological obstacles
Pérès et al [31], 2021	Older adults with a digital device	Digital intelligent platform available on smartphone, tablet, or computer and a VA	Home	Study protocol	Program impact and effectiveness; technical use; intervention mechanisms, transferability, and scalability conditions; health care consumption and outcomes; perceived social support; quality of life; loneliness; participation; sense of usefulness; self-esteem; frailty; activity limitation; program’s impact on health and care trajectories	N/A^j^
Pradhan et al [32], 2019	A total of 7 older adults (6 female adults and 1 male) aged >65 (mean 71.7) y with low technology use	VA commercial device (Amazon Echo Dot) with a paired tablet (Amazon Fire tablet)	Home or older adult living community	Qualitative (pre-post study)	Initial perceptions of VA technology, device perceptions and use, technology desired use, daily diary entries, voice commands use logs, and participants’ ontological categorization of VAs	Inconsistent use of personal pronouns for VA, users polite behaviors, users’ perceptions of VA did not clearly classify into “humanlike” or “object-like,” and VA role moving through different ontological categories
Razavi et al [33], 2022	A total of 19 older adults (13 female adults and 6 male adults) aged >60 (mean 71) y, with mild difficulties on social skills, depression and anxiety symptoms, and nonverbal impairment	Web-based automated version of a VA designed to improve communication skills	Home	Mixed (randomized controlled trial)	Verbal and nonverbal behavior in social communication (SSPA^k^); dialogue content	Significant improvement in eye contact and facial expressivity, users on average tend to provide longer responses as they proceed in a conversation, topic classes significantly affect users’ response length, and user sentiment significantly more positive for some topics than others
Reis et al [34], 2018	Older adults	VA commercial devices (Google Assistant, Amazon Alexa, Apple Siri, and Microsoft Cortana)	Older adult care center	Quantitative (VAs test)	VAs performance on acknowledgment, engagement, effectiveness, usefulness, and follow-up in 4 types of interaction (basic greeting, email management, social media, and social games)	VAs obtained good results in the acknowledgment and engagement, mixed results in effectiveness and usefulness, and bad results in follow-up (except for social games)
Simpson et al [35], 2020	Medical community, 2 older adults, adults	VA embodied as a household potted flower	Home or retirement home	Conference speech on design-thinking approach	Physical and mental health challenges that a VA could help mitigate; older adults’ everyday life, challenges, social interaction, thoughts on Vas, and their possible use; and device approachability and improvement	Development of device prototype
Striegl et al [36], 2021	A total of 9 older adults (2 female adults and 7 male adults) visiting day-care centers, without dementia (test with older adult participants); 4 professionals caregivers working in a day-care facility (2 female adults and 2 male adults) with age ranging from 33 to 61 y who had experience in caring for people with dementia	VA developed using the Amazon Alexa platform and Alexa Voice Services	Day-care facility	Mixed (usability test)	System usability, concept feasibility (5-point Likert scale), and participants technology affinity (test with older adults); task execution time, task number of interaction, task number of mistakes, system feasibility and usefulness (5-point Likert scale), opinion on new technology in the work environment, and technology affinity (test with caregiver)	High interest in talking to the VA and its functionality, high system feasibility to support people with dementia in ADL^l^, and step-by-step instructions perceived as useful (test with older adults); high system feasibility to support people with dementia in ADL, step-by-step instructions perceived as useful, appreciation for personalization option, user interface perceived as effective and motivating, mixed results on efficiency, and high results on efficiency and effectiveness (test with caregiver)
Torres et al [37], 2018	Older adults	VA	Home	Conference speech on project	Definition of project objectives, scientific and technological goals, and actions	N/A

^a^VA: voice assistant.

^b^PACS: postacute COVID-19 syndrome.

^c^HRQoL: health-related quality of life.

^d^DASS-21: Depression Anxiety Scale-21.

^e^CD-RISC-25: Connor-Davidson Resilience Scale-25.

^f^EQ-5D-5L: EuroQol-5 Dimensions-5 Levels.

^g^ISI: Insomnia Severity Index.

^h^SF-36: 36-Item Short Form Health Survey.

^i^NGD: normalized Google distance.

^j^N/A: not applicable.

^k^SSPA: Social Skills Performance Assessment.

^l^ADL: activities of daily living.

### Bibliometric Analysis

A bibliometric analysis was also conducted to construct a map of the selected articles using VOSviewer software (version 1.6.19; Leiden University’s Centre for Science and Technology Studies). This tool represents one of the most popular programs for bibliometric cluster mapping [38].

To illustrate the keyword co-occurrence network, keywords were extracted from the list of the 16 included articles.

During the map creation, the authors choose the co-occurrence type of analysis on keywords and selected full counting as the counting method. The threshold of the minimum number of occurrences of a keyword was set at 2 keywords. All the keywords were illustrated regardless of the greatest total link strength. At the selected keywords’ verification step, the authors considered it convenient to merge similar words by creating a thesaurus file. Thus, the thesaurus file included a column of similar keywords and another column with the keyword to be replaced with. Hence, in the final step, the selected keywords were analyzed using the Association Strength normalization method and default values. In addition, for clustering, the default values of resolution (ie, 1.00), minimum cluster size (ie, 1), and merge small cluster option were used.

## Results

In the following sections, the synthesis of the findings and results of the bibliometric analysis and qualitative scoring of the 16 selected articles are presented.

### Synthesis of Findings

The selected articles were assessed with regard to population, technological solution, environment, study design, outcomes, and main results. Table 1 presents a synthesis of the findings.

### Population

In summary, the population most frequently involved in the selected studies is older adults. In some cases, informal caregivers [22], geriatric experts [29], the medical community, the general public [35], or formal caregivers working in a day-care facility with experience in caring for people with dementia [36] are also involved. All the articles detail the total number of people engaged, except for 31% (5/16) of the articles [26,31,32,34,35]. The remaining articles involve a minimum of 7 and a maximum of 109 older adults. Among the selected articles, the age of the population varies widely, including people aged >50 [22,24,26,30], >60 [23,33], >65 [27,29], and >75 years [28]. Naturally, professionals are younger, ranging from 21 [29] to 33 [36] years. However, for some articles [25,31,34-37], there is no information on the age of the population involved. Instead, the sex of the participants is only specified in 56% (9/16) of the articles [24,26-30,32,33,36], in which a majority of female users are included.

In addition, 25% (4/16) of the articles consider participants’ familiarity with technology, involving only people with no experience with VA technology [26] and digital devices [31], involving only people with low technology use [32], or specifying people’s technological abilities [27]. In addition, some studies consider clinical conditions: 6% (1/16) of the articles [22] included people with diabetes or long-term health conditions, whereas others include people with postacute COVID-19 syndrome [24]; with normative cognitive functioning [28]; with no severe visual or hearing impairment and no moderate to severe cognitive impairment [30]; with mild difficulties in social skills, depression and anxiety symptoms, and nonverbal impairment [33]; and without dementia [36].

### Technological Solution

Regarding VA technology solutions, 44% (7/16) of the articles [22,24,25,28,29,32,34] report the use of commercially available VAs, for example, Google Assistant, Amazon Alexa, Apple Siri, and Microsoft Cortana. Some studies specify the design of new VA systems developed using the Amazon Alexa platform and Alexa Voice services [36] or implementing the Google Voice Android Software Development Kit on a tablet [27]. In other studies, the newly designed VA is embedded in a mobile app [23], a PC application [26], or even embodied as a household potted flower [35]. A total of 13% (2/16) of the articles [31,32] describe the design and the testing of a new VA-based digital intelligent platform. Finally, 1 (6%) article [33] presents a web-based automated version of a VA designed to improve communication skills, whereas another one [37] involves a personalized and expressive VA.

### Environment

The environment in most of the articles [22,24,25,29-31,33,37] is the home, which is alternated, in the study by Pradhan et al [32], with the older adult living community and, in the studies by Bravo et al [23] and Simpson et al [35], with the retirement home. Instead, the environments in other articles are the laboratory [26,27], the independent living facility [28], the older adult care center [34], and the day-care facility [36]. Thus, the selected articles concerning the use of a VA for social isolation and loneliness address both older adults living independently at home and those living in a facility.

### Study Design

Regarding the study design, among the 16 selected studies, 4 (25%) are quantitative, including 1 (6%) evaluation test [23], 1 (6%) pre-post study [24], 1 (6%) development and user test [26], and 1 (6%) VAs test [34]. Qualitative studies include 1 (6%) service evaluation [22], 1 (6%) evaluation test [29], and 1 (6%) pre- post study [32]. Then, there are 5 (31%) mixed studies, including both qualitative and quantitative methods, of which 1 (6%) is an evaluation test [27], 1 (6%) is a single-group quasi-experimental study [28], 1 (6%) is a pre-post study [30], 1 (6%) is a randomized controlled trial [33], and 1 (6%) was a usability study [36]. Finally, the remaining studies include 1 (6%) mini review [25], 2 (13%) conference speeches [35,37], and 1 (6%) study protocol [31]. More detailed information on the methodology results is presented in the *Quality Scoring* section.

### Outcomes

Among the outcomes, only 31% (5/16) of the articles [22,25,28,31,35] consider loneliness or social isolation. Of these 16 studies, only 1 (6%) [28] uses a standardized instrument—the 8-item University of California, Los Angeles (UCLA) Loneliness Scale—to assess the perception of loneliness. Instead, most articles (9/16, 56%) [22-24,27,29,30,32,35,36] focus on topics related to the acceptability, user experience, satisfaction, and usability of the technological solution, whereas a smaller number (2/16, 13%) [26,34] focuses on its technical performance. To evaluate these aspects, 5-point Likert scales are used only by 19% (3/16) of the articles [23,27,36].

Further outcomes addressed are verbal and nonverbal behavior in social communication [33], definition of project objectives, scientific and technological goals and actions [37], program impact on health and care trajectories [31], codes and overarching themes [29], interaction anthropomorphic aspects [28], and psychological and physical aspects such as frailty and quality of life [24,31].

### Main Results

Turning to the main results of using a VA, the impact on loneliness and social isolation is positive, leading to an improvement in users’ perceptions. Specifically, the participants in 13% (2/16) of the studies [22,24] report that the VA helped them cope with loneliness, whereas another study (1/16, 6%) [28] finds a significant reduction in perceived loneliness after 4 weeks of use and that the relational greetings from the user to the VA predict this reduction. Moreover, the loneliness experienced by the person forecasts the number of greetings he or she makes to the VA. Finally, a mini review (1/16, 6%) [25] outlines that the use of VA in older adults improves social connectedness and reduces loneliness.

Other benefits obtained include a positive impact on health and social well-being [22]; improvement in postacute COVID-19 syndrome symptoms, frailty, and health-related quality of life at 6 months follow-up [24]; sedentary life changes [24]; and significant improvement in eye contact and facial expressivity [33].

Regarding the VA, it is considered useful [24], satisfying [23,27], and interesting [36], and it obtains good results in the acknowledgment (the ability to recognize user contextual information) and engagement (the ability to maintain a coherent conversation) performance [34]. In addition, among participants in the study by Pech et al [30], 63% have a positive opinion toward the system used, and in the study by Striegl et al [36], both older adults and formal caregivers describe that the VA used have a high feasibility to support people with dementia in activities of daily living.

The main results also include technical information about the VA. For example, in 1 (6%) study [26], the VA obtains, in all the commands, a right answer ratio percentage >75%; another (1/16, 6%) study [29] identifies 8 major themes as possible VA beneficial functions; and another (1/16, 6%) study [32] presents crucial information for VA development, whereas in another (1/16, 6%) study [35], the device prototype is developed. Finally, critical issues emerge: VA interruptions when the person pauses for too long [27], older adults’ resistance to change, unplanned workload for a formal caregiver, specific technological obstacles [30], and bad results in the ability to suggest and perform some related activities at the end of the interaction [34]. Instead, the proposed improvements include facilitated access to professionals, communication at community events, late-night pharmacy service, customized activity proposals, and videoconferencing [30].

For 13% (2/16) of the articles [31,37], it is not applicable to define the main results.

### Bibliometric Analysis

Along with the bibliometric analysis, the authors built a thesaurus file containing the words that can be replaced, considering their very close meaning. The thesaurus file is presented in Table 2.

The bibliometric analysis extracted 221 keywords from the included articles, of which 36 (16%) met the threshold of 2 occurrences. The keyword list is presented in Table 3, in descending order of occurrence, showing the number of occurrences and the total link strength.

As can be observed in Table 3, the most used keywords by occurrence were as follows: “social isolation” (n=8), “human” (n=6), “older adults” (n=6), “aged” (n=5), “covid-19” (n=5), “loneliness” (n=5), “human computer interaction” (n=4), and “voice assistant” (n=4).

The most used keywords by total link strength, as shown in Table 3, were as follows: “human” (n=53), “aged” (n=44), “loneliness” (n=44), “social isolation” (n=42), “covid-19” (n=42), “pandemics” (n=29), “very elderly” (n=29), “older adults” (n=28), “prospective study” (n=25), “quality of life” (n=25).

**Figure 3:**
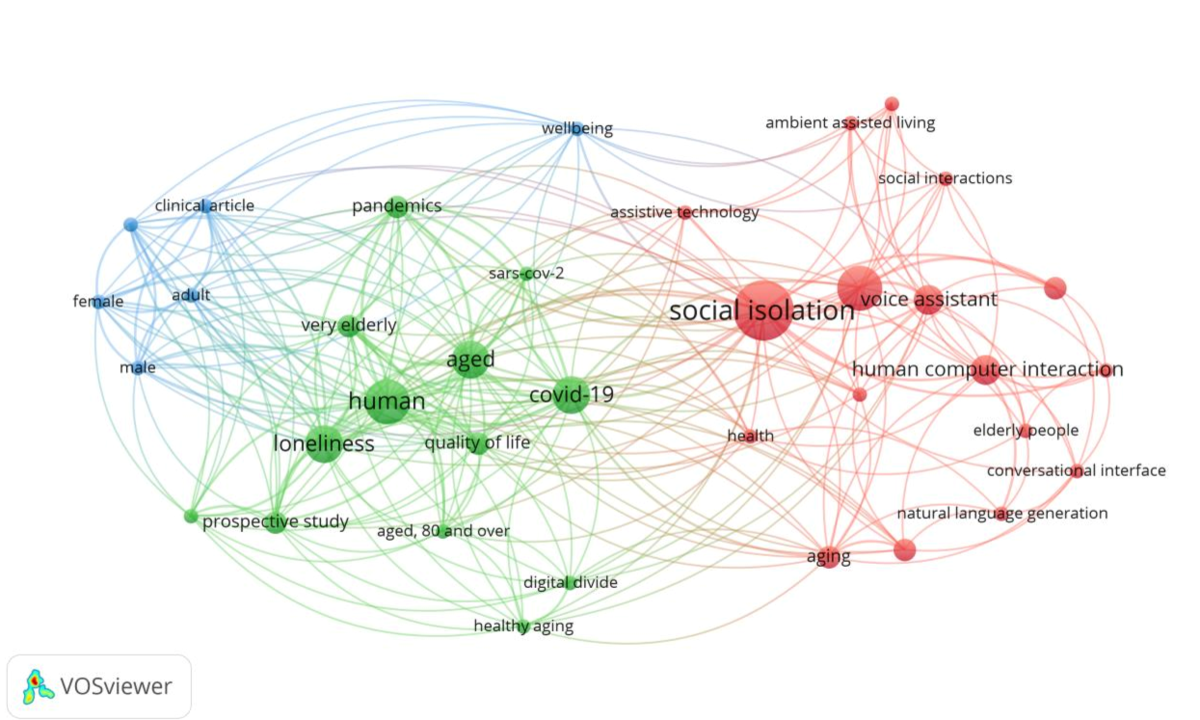
Bibliometric network visualization.

The bibliometric network is illustrated in Figure 3 and consists of 3 clusters of 36 keywords. The clusters are presented in more detail in Table 4, where each keyword from a cluster is shown in descending order by occurrence.

**Table 2:** Keywords merging using the thesaurus file.

Label	Replace by
human-computer interaction	human computer interaction
humans	human
pandemic	pandemics
Prospective studies	Prospective study
Voice assistants	Voice assistant

**Table 3:** The list of keywords, number of occurrences, and total link strength. Keywords are presented in descending order of occurrence.

Keyword	Occurrences	Total link strength
social isolation	8	42
human	6	53
older adults	6	28
aged	5	44
covid-19	5	42
loneliness	5	44
human computer interaction	4	16
voice assistant	4	18
aging	3	17
artificial intelligence	3	12
conversational agents	3	10
pandemics	3	29
prospective study	3	25
quality of life	3	25
very elderly	3	29
adult	2	21
aged, 80 and over	2	17
ambient assisted living	2	9
anthropomorphism	2	11
assisted living	2	9
assistive technology	2	13
clinical article	2	21
clinical assessment	2	17
conversational interface	2	8
digital divide	2	14
elderly people	2	7
female	2	21
health	2	12
healthy aging	2	14
male	2	21
middle aged	2	21
natural language generation	2	7
sars-cov-2	2	17
social interactions	2	9
user interfaces	2	8
well-being	2	17

**Table 4:** Keywords clustering.

Cluster and keywords			Occurrences		Total link strength
**Cluster 1 (red color; size: 17 items)**					
	social isolation	8		42	
	human	6		53	
	older adults	6		28	
	human computer interaction	4		16	
	voice assistant	4		18	
	aging	3		17	
	artificial intelligence	3		12	
	conversational agents	3		10	
	ambient assisted living	2		9	
	anthropomorphism	2		11	
	assisted living	2		9	
	assistive technology	2		13	
	conversational interface	2		8	
	elderly people	2		7	
	natural language generation	2		7	
	social interactions	2		9	
	user interfaces	2		8	
**Cluster 2 (green color; size: 13 items)**					
	aged	5		44	
	covid-19	5		42	
	loneliness	5		44	
	pandemics	3		29	
	prospective study	3		25	
	quality of life	3		25	
	very elderly	3		29	
	aged, 80 and over	2		17	
	clinical assessment	2		17	
	digital divide	2		14	
	health	2		12	
	healthy aging	2		14	
	sars-cov-2	2		17	
**Cluster 3 (blue color; size: 6 items)**					
	adult	2		21	
	clinical article	2		21	
	female	2		21	
	male	2		21	
	middle aged	2		21	
	well-being	2		17	

### Quality Scoring

According to the scoring tool, 13% (2/16) of the documents were assessed as being of poor quality in terms of the methodology. In the study by Simpson et al [35], it is unclear what the methodological information is based on, how it is presented, and if it is in line with other sources. The document is based on a conference speech on methods for the design-thinking approach. Instead, in the study by Torres et al [37], most of the information is not clearly sourced; it is unclear what the methodological information is based on and if it is in line with other sources. In addition, this paper is based on a speech at a conference on the objectives, goals, and actions of a research and innovation project.

A total of 56% (9/16) of the documents were considered medium quality. Specifically, 44% (7/16) articles [22,23,25-27,29,31] contain clear sources, methodological quality, and information value, presenting findings in line with the literature. Nevertheless, study designs were not of very high quality, representing mostly multiple case reports and case studies, whereas the study by Corbett et al [25] is a literature review.

A total of 13% (2/16) of the articles [24,34] have instead a more rigorous approach in the study design, representing a qualitative study and a single-group quasi-experimental study, respectively. However, the former is an abstract document lacking bibliographic references, while in the latter, it is unclear what the methodological information is based on. In both cases, the information presented is not clearly linked with the literature findings.

Finally, 31% (5/16) of the documents were deemed of high quality, considering that the information presented and the methodological information are clearly referenced. Among these, 1 (6%) article [33] is a randomized controlled study, while the remaining 25% (4/16) [28,30,32,36] are descriptive or observational studies.

Multimedia Appendix 2 [22-37] provides details of the quality scoring performed on the selected articles.

## Discussion

### Principal Findings

The purpose of this study is to synthesize knowledge about the use of VAs to reduce loneliness and social isolation among older adults.

Initially, after conducting the literature research, the quality of the selected articles is investigated, focusing on the strengths and weaknesses of the methodologies used. Of the 16 articles included in the review, only 2 (13%) articles [35,37] are considered poor quality, 9 (56%) articles [22-27,29,31,34] are medium quality, and 5 (31%) articles are high quality [28,30,32,33,36]. In summary, although recent publications in the literature on the use of VA by older adults for the reduction of loneliness and social isolation are not numerous, most of them are of medium to high methodological quality in terms of study design, authenticity, clear methodological quality, clear informational value, and representativeness of available primary sources.

After assessing the methodological quality of the selected articles, the findings are summarized, focusing on population, technological solution, environment, study design, outcomes, and main results for a more detailed overview. Among the 16 articles presented, most focus on the evaluation of acceptability, user experience, satisfaction, usability, or performance of the VA, while only 5 (31%) papers deepen the theme of social isolation and loneliness. Of these studies, 1 (6%) [31] has no available results, as it is a study protocol, and another (6%) [35] reached the development stage of a VA prototype. Therefore, 3 (19%) articles remain that investigate the possible effect of the use of a VA on social isolation and loneliness by older adults.

The first paper [22], a service evaluation study, found that using a VA for 2 months at home helped people with diabetes or other long-term health conditions (such as multiple sclerosis, dementia, and depression) combat loneliness. This is particularly relevant because it seems that social isolation increases the risk of mortality through physiological upregulation of chronic inflammation. This impact is significant even for middle-aged people, but is greater for older adults, particularly men [39]. Thus, the results obtained from the use of VAs are particularly relevant considering the population the study targeted but an assessment of loneliness would be needed to investigate the actual impact of the use on this dimension.

The second paper, a single-group quasi-experimental study [28], reported a significant reduction in perceived loneliness, assessed through the 8-item UCLA Loneliness Scale, after older adults living in an independent living facility used a VA for 4 weeks. Thus, loneliness among older adults living alone using a VA has decreased. Moreover, the loneliness perceived at the beginning of the intervention by participants predicts the number of greetings to the VA (such as “Good morning” or “Alexa, I’m home”), and, in addition, these relational greetings forecast loneliness reduction during the month of use. Therefore, according to the authors, VA anthropomorphization might have a role in combating loneliness in older adults.

Finally, the results of a mini review [25] suggest that the VA reduces loneliness among older adults and increases their connectedness. Older adults perceive the VA as a “companion,” especially those who live alone or have solitary lives for most of the day.

These studies show encouraging results about the potential of a VA in reducing social isolation and loneliness in older adults, in line with the suggestion from a systematic review [40] that new technologies can be promising opportunities to reduce social isolation and loneliness in this population. For example, 1 (6%) study found that the use of technology by older adults predicts less loneliness, which has in turn been associated with, on the one hand, better self-reported health and subjective well-being and, on the other hand, fewer chronic diseases and less depression [41]. Therefore, these are preliminary results suggesting that the association between technology use and physical and mental health may be mediated by loneliness.

VAs have the potential to be used by older adults to reduce their social isolation and loneliness, and the results presented go in that direction; however, they cannot be exhaustive nor conclusive.

Finally, the bibliometric cluster mapping analysis provides valuable insights into the relationships between keywords in the included articles. The generated keyword co-occurrence network revealed 3 distinct clusters, each representing a specific theme or concept in the literature.

Cluster 1, represented by keywords such as “social isolation,” “elderly people,” “voice assistant,” and “human computer interaction,” highlights the relevance of VA technology in combating social isolation among older adults. This cluster emphasizes the relevance of the topic. A VA could be a promising tool for facilitating social interactions, promoting well-being, and addressing the challenges faced by older people regarding social isolation. The relevance of VAs in addressing social isolation among older adults aligns with the findings of Portet et al [9] on the design and evaluation of a smart home VA for older adults. This cluster also corresponds to the author’s focus on the use of quality scoring to evaluate the methodological strengths and weaknesses of the studies, as the inclusion of studies exploring the effectiveness of VAs in combating social isolation would be of particular interest. This cluster emphasizes the importance of designing user-friendly interfaces and incorporating natural language generation and recognition for effective human-computer interaction. This cluster aligns with the literature on ambient assisted living, assistive technology, and artificial intelligence, and it is supported by the work presented in 1 (6%) article [10] on VAs and their applications, as well as in another (1/16, 6%) article [8] that discusses technological solutions for addressing social isolation and loneliness in primary care.

Cluster 2 emphasizes the significance of age in the context of loneliness. Keywords such as “loneliness,” “human,” and “quality of life” indicate the importance of understanding the psychological and emotional aspects of loneliness, considering the diverse experiences of individuals across different demographics. This is supported by the works presented by Valtorta and Hanratty [3] and Holt-Lunstad et al [7], who discuss the association between loneliness, social isolation, and health outcomes in older adults, emphasizing the importance of considering demographic factors in understanding and addressing these issues. Cluster 2 is also relevant in the context of the COVID-19 pandemic, as it includes keywords such as “COVID-19,” “pandemics,” and “digital divide,” which illustrates the impact of the pandemic on social isolation and the need for technological solutions, such as VAs, to bridge the digital divide and ensure connectivity and support for older adults during times of crisis. A study [6] on the association between social isolation, loneliness, and health outcomes in the context of coronary heart disease and stroke further emphasizes the significance of addressing social isolation during pandemics.

Cluster 3 encapsulates a range of keywords related to sex, clinical research, and well-being. The presence of keywords, such as “adult,” “female,” and “male,” along with “clinical article” and “well-being” underscores the importance of understanding how sex-specific factors can significantly impact overall well-being. This cluster likely refers to studies and investigations that explore the intersection of sex-related variables with clinical research outcomes, shedding light on how these factors can influence health and well-being differently among various demographic groups. Moreover, Cluster 3 may offer valuable insights into the evolving landscape of clinical research and its focus on addressing sex-specific health concerns, thus promoting a more comprehensive approach to well-being across diverse populations.

These clusters shed light on important topics related to social isolation, loneliness, and the use of VAs in addressing these issues among older adults. The findings underlined here can inform future research, interventions, and policy development in the field of geriatric care and technology.

### Strengths and Limitations

The study provides a comprehensive exploration of voice assistance systems used by older individuals, highlighting popular examples such as Amazon Alexa, Google Assistant, Apple Siri, Microsoft Cortana, Samsung Bixby, and Huawei HiVoice. The study examines the strengths and limitations of these systems.

One of the notable strengths of this study is its investigation into the use of VAs to alleviate loneliness and social isolation among older adults. This topic is fairly recent, but its relevance is growing in both the scientific and technological communities.

Moreover, this investigation is supported by both a literature review and a bibliometric analysis to gather as much knowledge as possible on the role of technology in combating loneliness and social isolation in older adults.

In addition, the selection of studies included in the article underwent an independent evaluation process by the authors, with any disagreements or uncertainties being resolved through discussion.

Another strength is the consideration of the scientific articles published in 2018. This choice was driven by the fact that VAs are relatively new and are continually advancing technological solutions. Furthermore, the application of such technology among older individuals is not yet widespread, resulting in a limited number of studies available on the topic. Despite this limitation, the potential benefits of VA solutions for older adults are highly intriguing, and this study aims to shed light on possible applications and the associated impact on older users.

This study also has limitations that need to be pointed out. First, the number of publications in the systematic review is reduced because the topic has only gained relevance recently. However, the authors decided to proceed with the bibliometric analysis to contribute in terms of interpretation, even though the number of papers on the use of VAs to reduce loneliness and social isolation among older adults is limited. Further limitations relate to the fact that 1 (6%) article [42] could not be retrieved and that the synthesis of findings is not comprehensive, as only the abstract was available for 1 article [24], nor complete, as it was not applicable to define the main results of 13% (2/16) articles [31,37]. Moreover, the selected studies had great heterogeneity, with only 6% (1/16) of studies [33] having a control group and 6% (1/16) of studies [28] having follow-up. Concerning the information about the population, it is not specified if people involved in the studies live alone or not. This could limit considerations regarding social isolation and loneliness. Finally, most articles collected qualitative data without providing quantitative instruments to assess the actual impact of VA use.

### Future Directions

On the basis of this literature review and bibliometric analysis, several priorities for future research can be identified. First, working with keywords from clusters 1 and 2, it is easy to see that “loneliness” and “social isolation” have a huge impact on older people [43]. On the basis of our literature review, authors are more interested in system use and acceptability [30], acceptance user experience [22], and system usability [36], which are just some examples. The main points are “loneliness” and “social isolation,” and we only found 1 study [28] to reduce perceived loneliness in older adults. Thus, the topic of the use of VA for social isolation and loneliness among older adults seems to be underestimated in comparison to user experience aspects, which are more deeply investigated in the scientific literature.

Similarly, we encourage that researchers include questionnaires to measure loneliness in future studies, for example, the Revised UCLA Loneliness Scale [44], the De Jong Gierveld Loneliness Scale [45,46], the Steptoe Social Isolation Index for social isolation [44], and the Cornwell Perceived Isolation Scale for perceived isolation [47], for use with VA systems based on artificial intelligence techniques or other related systems to improve the life expectancy of older people. For other specific information about these questionnaires, refer to *Social Isolation and Loneliness in Older Adults: Opportunities for the Health Care System* [48]. Second, this work shows that the terms social isolation and loneliness are still often treated as interchangeable, although they are actually related but distinct concepts [3].

In fact, nowadays, the tendency is to refer to loneliness as a subjective negative feeling of perceiving a lack of social network or desired companion, whereas social isolation is the objective lack or scarcity of social contacts and interactions with family, friends, or community [3]. Therefore, it would be particularly relevant if future studies would clearly define which dimensions they measure, as mentioned in the preceding section. Third, future research should examine the large heterogeneity within the older adult population. Some of the selected articles described different characteristics of the population, but none delved into the possible different impacts of VA use in relation to these variables. Future studies should explore the effects of using a VA on the social isolation and loneliness of older adults, investigating possible differences in sex, socioeconomic background, and also familiarity with technology and living conditions.

### Conclusions

This paper conducted a literature review and a bibliometric analysis of the use of VAs among older adults to reduce social isolation and loneliness. The findings indicate that most studies focus on the usability, acceptability, or user experience of the VA. However, studies directly addressing the impact that using a VA has on the social isolation and loneliness of older adults have positive results and provide important information for future research, interventions, and policy development in the field of geriatric care and technology.

## Appendix

Multimedia Appendix 1 Excluded articles and motivations for the exclusion.

Multimedia Appendix 2 Quality scoring of selected articles.

## Abbreviations

PRISMA: Preferred Reporting Items for Systematic Reviews and Meta-Analyses
UCLA: University of California, Los Angeles
VA: voice assistant
