# Supplementary material for: Reducing Loneliness and Social Isolation of Older Adults Through Voice Assistants: Literature Review and Bibliometric Analysis
Source: J Med Internet Res. 2024 Mar 18;26:e50534. doi: 10.2196/50534 (PMC10985600; doi:10.2196/50534)
Supplement: Multimedia Appendix 1 [file jmir_v26i1e50534_app1.docx]

**Multimedia Appendix 1** **Excluded articles and motivations for the exclusion**

| **Reference** | **Motivation** |
| --- | --- |
| Chen et al, 2020 [12] | Vocal assistant (VA) application (Mobile app-based system) |
| Eimontaite et al, 2020 [13] | Different technology used (Autonomous driving simulator trial with vocal assistant) |
| Eirale et al, 2022 [14] | Different technology used (Robotic Assistant) |
| Martin-Hammond et al, 2019 [15] | Marginality of VA topic (Focus on general aspects of Intelligent Assistant) |
| Méndez et al, 2020 [16] | Different technology used (Smart home and multi-sensor system) |
| Restyandito et al, 2020 [17] | VA application (Social media application interface) |
| Syeda et al, 2019 [18] | Different technology used (Messenger based voice message service) |
| Zhou et al, 2022 [19] | Different technology used (Social robots to assist older people with dementia) |
