# Supplementary material for: Reducing Loneliness and Social Isolation of Older Adults Through Voice Assistants: Literature Review and Bibliometric Analysis
Source: J Med Internet Res. 2024 Mar 18;26:e50534. doi: 10.2196/50534 (PMC10985600; doi:10.2196/50534)
Supplement: Multimedia Appendix 2 [file jmir_v26i1e50534_app2.docx]

**Multimedia Appendix 2** **Quality scoring of selected articles**

| **Reference** | **Part 1 Criteria** | **Part 2 Criteria (Point 1)** | **Part 2 Criteria (Point 2)** | **Part 2 Criteria (Point 3)** | **Part 2 Criteria (Point 4)** | **Part 2 Criteria Total** | **Total score** |
| --- | --- | --- | --- | --- | --- | --- | --- |
| Balasubramanian et al, 2021 [23] | 3 | 1 | 1 | 1 | 1 | 4 | 7 |
| Bravo et al, 2022 [24] | 3 | 1 | 1 | 1 | 1 | 4 | 7 |
| Caselgrandi et al, 2021 [25] | 5 | 0 | 1 | 1 | 0 | 2 | 7 |
| Corbett et al, 2021 [26] | 3 | 1 | 1 | 1 | 1 | 4 | 7 |
| Farías-Barraza et al, 2022 [27] | 3 | 1 | 1 | 1 | 1 | 4 | 7 |
| García-Méndez et al, 2021 [28] | 3 | 1 | 1 | 1 | 1 | 4 | 7 |
| Jones et al, 2021 [29] | 5 | 1 | 1 | 1 | 1 | 4 | 9 |
| O'Brien et al, 2022 [30] | 3 | 1 | 1 | 1 | 1 | 4 | 7 |
| Pech et al, 2022 [31] | 5 | 1 | 1 | 1 | 1 | 4 | 9 |
| Pérès et al, 2021 [32] | 2 | 1 | 1 | 1 | 1 | 4 | 6 |
| Pradhan et al, 2019 [33] | 5 | 1 | 1 | 1 | 1 | 4 | 9 |
| Razavi et al, 2022 [34] | 6 | 1 | 1 | 1 | 1 | 4 | 10 |
| Reis et al, 2018 [35] | 5 | 1 | 0 | 1 | 0 | 2 | 7 |
| Simpson et al, 2020 [36] | 2 | 1 | 0 | 0 | 0 | 1 | 3 |
| Striegl et al, 2021 [37] | 5 | 1 | 1 | 1 | 1 | 4 | 9 |
| Torres et al, 2018 [38] | 2 | 0 | 0 | 1 | 0 | 1 | 3 |
